# Difficult-to-treat asthma, is it really difficult?

**DOI:** 10.4103/1817-1737.74268

**Published:** 2011

**Authors:** Mohamed S. Al-Hajjaj

**Affiliations:** *Department of Medicine, Medical College, King Saud University, Riyadh, Saudi Arabia*

Difficult-to-treat asthma (DTA) carries several names; each one points to an aspect of the disease. Chronic severe asthma, steroid-dependent asthma, difficult-to-control asthma, and refractory asthma are some of these terminologies.[1] Causes of DTA can be divided into genuine and nongenuine etiologies. Nongenuine causes can be further divided into three categories: (1) misdiagnosis where the problem is not bronchial asthma to start with, but another respiratory system pathology that is not appropriately addressed, e.g. bronchiectasis, endo-bronchial tumors, and vocal cord dysfunction,[2] (2) comorbidity that worsen bronchial asthma and making it difficult to manage, e.g. chronic sinusitis, gastro-esophageal disease, sleep apnea syndrome, and congestive heart failure (CHF),[3] and (3) confounding factors, e.g. nonadherence with treatment, the presence of allergens at home or work and psychosocial problems making asthma difficult to treat.[4–6]

After dealing with nongenuine causes that are making asthma difficult to treat, we will be left with asthmatic patients who have a real problem of not responding to steroid therapy (the genuine type). Patients may differ in the degree of this phenomenon of “steroid unresponsiveness”. Some of them may have the “pseudo-steroid” resistance status which is due to other coexisting conditions,[7] others do not respond adequately to high doses of inhaled steroids but will need continuous oral therapy to show a reasonable response.[8,9] In my practice, I have not seen any patient with total unresponsiveness to oral steroids. Hence, my own definition for DTA is “asthmatic patients in whom it is not possible to take off oral steroids”. These patients are having persistent symptoms despite high doses of inhaled steroids and other “nonsteroidal” asthma therapy.[10,11]

Asthma in general is a chronic disease that can be triggered by many factors such as allergens, irritants, infections exercise, stress, and neurogenic or hormonal factors.[11] It is characterized pathogenically by peculiar inflammatory reaction and variable bronchospasm, and clinically by characteristic symptoms, the most common of which are shortness of breath; cough with or without expectoration, wheezing and tightness of the chest. These symptoms can interfere with the patients’ everyday activity, reduce productivity, and result in poor quality of life.[12] Therefore, the objectives of asthma management is to prevent and relief these symptoms, as well as achieving a normal patient’s daily activity. In DTA, this is very difficult to achieve and therefore the aim of its treatment is to reach the best possible outcome; that is, as few symptoms and exacerbations as possible. DTA has a more significant burden in terms of healthcare-related expenses, decreased productivity, and reduced quality of life for patients.[13] Although these patients make about 5–10% of all asthmatics, they account for close to 50% of the total cost of asthma therapy.[14]

Asthmatic patients with persistent symptoms despite adequate maintenance therapy should be systematically evaluated to identify factors contributing to the poor control and then adopt a targeted intervention scheme.[15,16] This includes a through history to identify precipitating factors, home or work allergens, compliance with medications, nature of their illness, and any other relevant issues. A complete physical examination for the chest and the rest of the body is to be done to explore other comorbidities such as the presence of signs of bronchiectasis, interstitial lung disease, CHF, and joint disease. Laboratory tests include complete blood count, serum levels of IgE, schistosoma serology, screening for liver and kidney diseases, and other inflammatory biomarkers if relevant and available.[17] Radiological tests include chest radiography, computerized chest, and sinuses scans. Other investigations are requested according to the clinical situation and differential diagnosis.[18]

DTA is currently the subject of extensive research worldwide. The most important question is that despite the availability of very effective drug therapy; what makes asthma in some patients difficult to treat?[19] There are many other unanswered questions, such as which patients will be responders or non--responders to steroids, and why and how to deal with the latter. These are some of the questions that need prospective, long-term studies to be clearly answered. The mechanism of steroid resistance is unknown, but it is possible that abnormalities in glucocorticoid receptor number, glucocorticoid receptor binding, or abnormalities in the glucocorticoid–glucocorticoid receptor complex binding to DNA are likely to account for the poor response to corticosteroid therapy in these patients.[20,21]

Therefore, to answer the question in the title is to state that if a systematic approach is followed and all issues related to the problem of DTA are addressed, we will be left with only a small percentage of patients (5–10%) in whom a real problem of refractory asthma exists. A referral to an expert pulmonologist or a specialized center is advised where new therapies are available and experimental drug trials can be conducted.

In Saudi Arabia, patients with DTA are often overlooked and ignored. Many patients will be having frequent exacerbations and hospital admissions. Other will be put on continuous oral or even frequent intramuscular steroid injections. Another sizable group will seek nonconventional therapy with herbal weeds or nonmedical forms of therapies. They may wander from one clinic to another and end up on a long-term oral steroids with unsatisfactory results with all the known complications of corticosteroids. An excellence center as a referral for managing these patients is urgently needed.
